# Acceptability, feasibility and implementation of a web-based U.S. Health Insurance Navigation Tool (HINT)

**DOI:** 10.1186/s13104-021-05577-w

**Published:** 2021-05-01

**Authors:** Caiwei Zheng, Alberto J. Caban-Martinez

**Affiliations:** 1 Department of Public Health Sciences, Leonard M. Miller School of Medicine, University of Miami, Miller School of Medicine, 1120 N.W. 14th Street, Room #1025, Miami, FL 33136 USA; 2 Leonard M. Miller School of Medicine, Physical Medicine and Rehabilitation of the University of Miami, Miami, FL USA

**Keywords:** Health insurance, Insurance marketplace, Navigation tool, Healthcare access

## Abstract

**Objective:**

In the U.S., health insurance is a crucial determinant of the affordability of healthcare services and access to care. Population-based studies indicate Americans do not have an adequate understanding of their insurance plans and face difficulties navigating their health insurance coverage. The purpose of this pilot study is to collect qualitative data using a key informant interview format to learn about the acceptability, feasibility and implementation of a newly devised online health insurance navigation tool (HINT).

**Results:**

A total of 57 Florida residents completed the 18-item HINT web-based survey tool and provided feedback on their experience, of which 63.2% were women, 40.7% Black race, and had average sample age of 46.9 years. Participants reported the web tool to be of good length, easy readability, relevant, and overall helpful for insurance selection. All respondents reported that they would use the tool themselves should they find themselves in the process of selecting an insurance plan and 98.2% of respondents reported that they would suggest the web tool to a family or friend. The average time it took to complete the questionnaire was 4 min and 20 s.The HINT tool met study criteria on feasibility, implementation, and acceptability among study participants.

## Introduction

In the U.S., health insurance is a crucial determinant of the affordability of healthcare services and hence one’s access to quality care. Among individuals selecting a health care insurance plan, the process can be a daunting and complex task with significant financial and health consequences. An informed decision requires thorough understanding of the coverage of the specific plan options and accurate estimation of their potential health service spending. Study findings show that approximately one-half of U.S. adult consumers report having inadequate health insurance literacy (i.e. ability to seek, obtain, understand and use health insurance plans) and 48% have low confidence using their insurance to access health care services [1]. In a survey of 202 insured US adults, only 14% could answer 4 simple questions on the definition of cost-sharing features [2]. The lack of literacy and ability to navigate one’s own health insurance plans has direct and potentially devasting impact on the health of the public, with literature suggesting that low health insurance literacy may be associate with greater avoidance of both preventive and nonpreventive medical services [3].

Healthcare navigation tools aimed to aid consumer decision making exists but mainly targets the gap in understanding of insurance related terminology and plan features and have varying reliability. It has been reported that the internet, mail, and television are among the most frequently used resources for consumers seeking information on health insurance but study participants reported having low trust in all 3 sources [4]. Resources as such are also often overwhelmingly lengthy and the sponsored “tools” and myriad marketing efforts make reliable, impartial information hard to find. Even if impartial, accurate information is found, such consumer education efforts are unlikely to significantly improve decision making because they provide information with little guidance on its application. Use of plain language, presenting insurance terminology in context and organizing information to support its use in weighing tradeoffs between cost and coverage have been suggested to be potentially effective ways to improve decision-making.[5].

The Health Insurance Navigation Tool (HINT) pilot project sought to develop an innovative online health insurance navigation tool that evaluates the trade-off between cost-sharing features and premium costs for the consumer, taking in to account their healthcare expense estimate. An 18-item questionnaire was designed to elicit health insurance consumer information that could ultimately predict spending and utilization of services, as well as screen the consumer for their eligibility for special government programs as an alternative to commercial insurance. The HINTS algorithm would then generate a consumer report based on the answers given which compares the consumer’s compatibility with each of the four available major health insurance plan types: Health Maintenance Organization (HMO), Preferred Provider Organization (PPO), Exclusive Provider Organization (EPO), and Point of Service (POS). In the president study, we assess the acceptability, feasibility and implementation of the HINTS questionnaire among a sample of health insurance consumers.

## Main text

### Methods

#### Study design, participant recruitment and consent

We used a cross-sectional design to evaluate the acceptability, feasibility and implementation of a newly developed web-based Health Insurance Navigation Tool (HINT) among a convenience sample of health insurance consumers, in Florida, USA. Study participants were recruited between November 2018 to December 2020 from a variety of public places (i.e., city library, residential buildings, schools, hospitals etc.) and at local community events such as health fairs. All consenting individuals were 18 years of age and older, able to speak and read in English and of any race, ethnicity and social demographic background. Participants were fully explained the purpose of the study, asked any questions about the study, and subsequently completed a verbal consent process given no personal identifying information was collected by the survey instrument nor during the interview process. Recruitment of new participants was terminated when interview feedback reached saturation. A USB thumb drive on a lanyard was offered as a small incentive to participate.

### Study demographics survey and interview procedures

Once consented to participate, recruited community member was assigned a random study participant ID number to ensure anonymity in documentation. Participants were initially administered a socio-demographics questionnaire via Qualtrics. Participants were then asked to test out the online navigation tool independently using a provided computer and encouraged to make handwritten notes of any issues they encounter on a printed copy of the 18-item HINTS question survey instrument. After completion, the researcher interviewed the participant in a one-on-one format that was audio-recorded and later transcribed as needed for analysis. A semi-structured script with a list of questions about the feasibility, implementation, and acceptability of the HINTS instrument were discussed between the research team and participant (Table 1).

**Table 1:** Metrics used to assess feasibility, acceptability, and implementation Health Insurance Navigation Tool (HINT) pilot project

Metric	Key questions	Data source	Indicators	Results
Feasibility	Is the web-based navigation tool of reasonable length?	Key informant interview	Percentage of participants who are satisfied with the length of the assessment	83.9% (8.9% missing)
	Are consumers capable of completing the web-based navigation tool independently to receive a result?	Key informant interview	Percentage of study subjects who complete the web assessment without asking for assistance	93.0%
	Are questions in the HINT tool easily understandable?	Key informant interview	Percentage of study subjects who report good readability	89.3% (7.1% missing)
Acceptability	Are health insurance consumers willing to use a web-based healthcare navigation tool?	Key informant interview	Percentage of study subjects who report that they would use the HINT tool when selecting a health insurance	100.0%
	Do consumers find this navigation tool helpful in selecting an insurance?	Key informant interview	Percentage of study subjects who report that they would recommend this tool to a friend or loved one in need	98.2%
	Do consumers find the navigation tool questions relevant to their goal of selecting an insurance?	Key informant interview	Percentage of study subjects who report that the questions are relevant	85.7% (14.3% missing)
Implementation	Are consumers comfortable with the online questionnaire format of the navigation tool?	Key informant interview	Percentage of study subjects who reported discomfort answering questions via the online tool	0.0%
	Can the web assessment be completed with ease?	Recorded time	Average time it takes to complete the 18-question web assessment	4 min 20 s
	Is the HINT tool accessible to the public?	Key informant interview	Percentage of study subjects who can navigate the web tool using a computer without asking for assistance	93.0%

#### HINTS instruments design

The HINTS survey is designed to streamline the health insurance selection process by reducing the complex terminology associated with health insurance plans to short, easy-to-understand questions that promote weighing the trade-off between cost and coverage. The goal is to help consumers make value-consistent decisions that are also financially sustainable. The HINTS survey consists of 18 multiple choice questions including: 3 questions regarding consumer healthcare budget, 7 questions on healthcare preferences, 6 questions that help predict healthcare utilization, and 2 questions that screen for eligibility for Medicaid (Table 2). Question answer choices are designed to represent the most salient features of the four major insurance plan types. And the algorithm determines compatibility by comparing the user’s answer choices to the answer combinations that best fit each of the plan types.

**Table 2:** Health Insurance Navigation Tool (HINT) Questionnaire Questions

Question category	Question number	Questions
Medicaid Screening	1	Do you make below $40,000 a year? Yes No
	2	Are you a U.S. Citizen or Permanent Resident? Yes No
Healthcare Utilization	3	When would you go to the doctor's office? I almost never go to the doctor I only go to the doctor when I feel seriously sick I go to the doctor when I have questions or concerns I go to the doctor regularly to prevent getting sick
	4	When I feel ill, I tend to: Brush it off if I can Call my family doctor's office Look for a doctor online to make an appointment
	5	When I decide NOT to go to the doctor though I feel ill, it is mostly because: The cost of the visit I do not have time I cannot easily make an appointment I do not feel like I need to
	6	(Pick the option that would most heavily influence your decision) I am more likely to go to the doctor if: The visits are more affordable Less paperwork is needed My plan does not restrict my choice of doctors None of the above will make it more likely for me to go to a doctor
	14	Preventive care is: Very important Optional Not important/I don't know what that is
	15	Regarding pharmacy use, select the option below that most accurately fits you: I use the pharmacy a lot because I take medications frequently I go to the pharmacy occasionally, when my doctor prescribes medication for me I almost never go to the pharmacy
Budget	7	I am willing to pay more than my ideal budget monthly for: More doctors to choose from Better coverage of individual visit costs with Primary Care doctors Better coverage of individual visit costs with Specialty doctors I would not be willing to pay over my ideal budget
	8	In addition to my monthly insurance payments, I am willing to spend an additional ______ per year out of pocket for doctor's visits and services: Less than $200 Up to $500 More than $500
	18	I am willing to spend ______ on my health insurance plan, per month: Up to $200 Between $201 and $400 More than $400
Healthcare Preferences	9	Select the option below that most accurately fits you: I would prefer to go to one general doctor for my health concerns and be referred to specialist doctors only when needed I would like to pick which doctor to go to based on my specific health concerns I don't have a preference/I don't know the difference
	10	In order to minimize your monthly payment, pick the factor more important to you: I would like to be able to choose my family/ primary care doctor I would like to be able to choose my specialists I don't know what the difference is/No preference I would be willing to pay more monthly for more flexibility in choosing both
	11	Regarding doctor choice, select the one option below that most accurately fits you: I have specific expectations for my doctor (in terms of education, training, background, experience etc.) and it can be hard to find doctors that satisfy them I have some specific expectations for my doctor and I can easily find doctors that can satisfy them I do not have expectations for my doctor other than he or she is licensed to practice
	12	Which of the following factors are important in dictating whether or not you will go to a doctor? Education/experience Location/availability Both A and B Neither A or B
	13	I would NOT go to a doctor that: (although covered by my insurance) requires payment that cost More than my budget Is difficult to make appointments with Is not covered by my insurance Has negative online reviews
	16	Select the option below that most accurately fits you: I am willing to switch to a different insurance plan to see a doctor(s) of my choice I am not willing to switch to a different insurance plan to see a doctor(s) of my choice Regarding coverage, which of the following most accurately describes you?
	17	Regarding coverage, which of the following most accurately describes you? It is important to me that my insurance covers most if not all my visits and treatments I would be okay with an insurance as long as it covers some of my visits and treatments I don't mind an insurance plan with less coverage as long as the monthly cost is low

#### Data analysis

We estimated frequency and descriptive statistics for all items available in the sociodemographic characteristics survey. Continuous variables such as age was expressed as mean ± SD, while categorical variables were expressed as frequency and percent. Statistical analysis of survey data was performed using the Statistical Package for the Social Sciences (IBM SPSS Statistics for Mac, version 24.0). The audio files collected during each key informant interview were transcribed by our study team and reviewed for accuracy by the research staff. A general inductive approach was used for data analysis. First‐ round coding was completed by two independent coders (CAZ and AJCM) and all transcripts were coded for primary codes. The coders then met to discuss their primary codes and reach a consensus in the definition and labeling of the code. Primary codes were then collapsed independently into secondary codes and the independent coders met again to reach a consensus of their secondary codes. Finally, secondary codes were collapsed into broader themes by the pair of coders.

### Results

In total, 57 participants completed both the demographics questionnaire and the key informant interview portions of this pilot study, with a moderate majority being women (Table 3). The majority of study participants were women (63.2%), Caucasian (45.8%), were college graduates (36.8%), never married (40.4%), identified English as their first language (66.1%), and 79.0% were the primary decision maker of health insurance in their household. The average age of the participants was 46.9 years of age with the youngest being 20 years of age and the oldest being 79 years of age.

**Table 3:** Sociodemographic characteristics among participants Health Insurance Navigation Tool (HINT) Study, (N = 57)

Characteristics	Average ± SD
Average age ± SD	46.9 ± 16.5
Gender	n (%)^a^
Male	21 (36.8)
Female	36 (63.2)
Race	
White	27 (45.8)
Black or African American	24 (40.7)
Asian	3 (5.1)
Native Hawaiian or Pacific islander	0 (0.0)
Other	5 (8.5)
Ethnicity	
Hispanic/Latinx	23 (41.1)
Non-Hispanic/non-Latinx	33 (58.9)
Highest educational attainment	
Never attended school/ kindergarten only	0 (0.00)
Grade 1–8 (elementary school)	1 (1.8)
Grade 9–11 (some high school)	3 (5.3)
Grade 12 or GED	15 (26.3)
Some college or technical school	10 (17.5)
College graduate	21 (36.8)
Master’s degree or higher	7 (12.3)
Marital status	
Married	17 (29.8)
Widowed	2 (3.5)
Divorced	11 (19.3)
Separated	2 (3.5)
Never married	23 (40.4)
A member of an unmarried couple	2 (3.5)
English as first language	
Yes	38 (66.1)
No	19 (33.9)
Primary household decision maker	
Yes	45 (79.0)
No	12 (21.0)

^a^Differences in sub-total population sample due to item non-response or missing

#### Feasibility, acceptability and implementation results

This study demonstrated that majority of the participant found the HINT tool to be of good length (n = 47, 83.9%), easy readability (n = 50, 89.3%), relevant (n = 48, 85.7%), and overall helpful for insurance selection.

To access feasibility, we asked participants to comment on the length of the HINT tool, for which they stated “The length is perfect in that it was enough questions that I could get like a thorough like idea of what I’m thinking about health insurance, but it was short enough that I didn’t feel overwhelmed or wanted to stop the assessment short” [Participant #1] and “ The length was fine, it was fairly comprehensive, and it asked a variety of questions, so I think it was…the length was sufficient to cover the questions that need to be asked” [Participant #40].

When asked to comment on the readability of the tool, one participant stated: “I thought it was pretty good, questions were straightforward, easy to read, easy to understand, it was nicely set up” [Participant #21]. And responses such as “It was easy to read and understand” [Participant #23], and “Your language is pretty self-explanatory” [Participant #41] were common.

In assessing acceptability, participants were asked for their general opinion on the HINT tool and stated: “It was very good, it was very effective”, “I thought that you touched on points that were very, uh very specific in the sense of a specialty doctor or primary and your pharmacy, things that you’d really look at when you’re looking for an insurance.” [Participant #36]. Another participant stated: “The explanation at the end I thought was very good, so I know what to go search for, that was clear.” and “Yea and I think this is valuable, because then I know I need to go look at POS, HMO or PPO plans, and I’m gonna look at those first, and then I can compare others to them, I can compare those together first and then I can compare others to them. And I think this is a great tool” [Participant #44] All respondents reported that they would use the tool themselves if they find themselves in the process of selecting an insurance plan and 98.2% of respondents reported that they would suggest the web too to a family or friend if they were in a similar situation.

For assessment of implementation, the average time it took to compete the web assessment was 4 min and 20 s. Of the 57 participants, 93.0% (n = 53) completed the questionnaire without asking for assistance. And participants stated: “I think it’s a good start, because having been on healthcare.gov there are question…but they don’t ask these particular questions, this feels a little bit more personal” [Participant #34], “It was nice, it was not labor intensive” [Participant #21] and “I though the questionnaire was very to the point, and easy to read and very uh…friendly” [Participant #36]. Based on data collected from key informant interviews, the HINT tool met study criteria on feasibility, implementation, and acceptability among study participants.

### Discussion

In this study, we asked a diverse group of participants to evaluate a novel healthcare insurance navigation tool. The majority of the participants reported no difficulty using the web-based tool and that the tool was easy to read, relevant, and of acceptable length. We found that the navigation tool took on average 4 min and 20 s for participants to complete independently. And our findings suggest that the HINT tool was feasible, accepted, and able to be successfully implemented by study participants. To our knowledge, there is currently no similar healthcare navigation tool reported by literature. We believe this study demonstrates the public’s need for such a resource for health insurance plan selection and provides support for this novel instrument to be implemented in healthcare navigation settings.

## Limitations

This study should be viewed in light of several limitations. First is the utilization of convenience sampling, which may have led to a disproportionate representation of population of lower socioeconomic status, as a majority of participants were recruited at local health fairs. However, it is important to note that this low-income sample of participants with restricted health care access was our primary target population whom we expect to benefit most from our web tool. Another limitation is the geographic location of sampling being in Florida. This led to a different racial diversity representation than that of the general population, as Florida is home to an older and often more racially/ethnically diverse population. The study overrepresents the black and Hispanic population and underrepresents the white population. A third source of bias is the use of a Mac computer to administer the web tool trial, which some participants expressed difficulty using. This may have led to an overestimate in time taken to complete the assessment. Despite these limitations, this study validates the need and acceptance for a health insurance guidance tool and supports its practical implementation. Future studies will benefit from testing this instrument in a wider population to identify subgroups of the population in which this tool is most acceptability and further improve its accessibility in various populations.

## Data Availability

The datasets generated during the HINT study are not publicly available due to the interview nature of the interview study, however de-identified data may be available from the corresponding author upon reasonable request.

## Abbreviations

EPO: Exclusive Provider Organization
HINT: Health Insurance Navigation Tool
HMO: Health Maintenance Organization
POS: Point of service
PPO: Preferred Provider Organization
USB: Universal serial bus
